# Antibacterial Activity and Biocompatibility with the Concentration of Ginger Fraction in Biodegradable Gelatin Methacryloyl (GelMA) Hydrogel Coating for Medical Implants

**DOI:** 10.3390/polym14235317

**Published:** 2022-12-05

**Authors:** Seo-young Kim, Ae-jin Choi, Jung-Eun Park, Yong-seok Jang, Min-ho Lee

**Affiliations:** 1 Department of Dental Biomaterials and Institute of Biodegradable Material and Oral Bioscience, School of Dentistry, Jeonbuk National University, Jeonju 54896, Republic of Korea; 2 Division of Functional Food & Nutrition, Department of Agrofood Resources, National Institute of Agricultural Science (NIAS), Rural Development Administration (RDA), Wanju-gun 55365, Republic of Korea

**Keywords:** surface modification, ginger fraction, gelatin methacryloyl (GelMA), sustained release, antibacterial effect, cytotoxicity, bone regeneration

## Abstract

The gingerols and shogaols derived from ginger have excellent antibacterial properties against oral bacteria. However, some researchers have noted their dose-dependent potential toxicity. The aim of this study was to enhance the biofunctionality and biocompatibility of the application of ginger to dental titanium screws. To increase the amount of coating of the n-hexane-fractionated ginger on the titanium surface and to control its release, ginger was loaded in different concentrations in a photo-crosslinkable GelMA hydrogel. To improve coating stability of the ginger hydrogel (GH), the wettability of the surface was modified by pre-calcification (TNC), then GH was applied on the surface. As a result, the ginger fraction, with a high content of phenolic compounds, was effective in the inhibition of the growth of *S. mutans* and *P. gingivalis*. The GH slowly released the main compounds of ginger and showed excellent antibacterial effects with the concentration. Although bone regeneration was slightly reduced with the ginger-loading concentration due to the increased contents of polyphenolic compounds, it was strongly supplemented through the promotion of osteosis formation by the hydrogel and TNC coating. Finally, we proved the biosafety and superior biofunctionalities the GH−TNC coating on a Ti implant. However, it is recommended to use an appropriate concentration, because an excessive concentration of ginger may affect the improved biocompatibility in clinical applications.

## 1. Introduction

Some antimicrobial agents (chlorhexidine, triclosan, cetylpyridinium chloride (CPC), etc.), which contain synthetic compounds, are commonly used to prevent dental caries [1,2]. Recently, with increasing concerns regarding the safety of synthetic compounds in the body, interest in natural products has gradually increased worldwide. Natural products are chemically and biologically diverse, and their unique natural compounds show low toxicity, as well as pharmacological activities such as anticancer, antibacterial, anti-inflammatory, and antioxidant effects [3,4].

Ginger, as one of the natural products, consists of monoterpenes, sesquiterpenes, diterpenes, vanilloids, flavonoids, etc. [5]. There are gingerols, shogaols, paradols, and zingerone, etc., in the main constituents of the nonvolatile pungent compounds present in ginger [6]. Because these specific compounds in ginger have excellent bioactivities, such as antibacterial, anti-inflammatory, antioxidant, and periodontal disease suppression effects, its extracts are used in aspects of the field of oral health care, including oral medicine and care products [7,8].

Titanium (Ti) implants have been used as a dental material for a long time. However, they pose a risk of the occurrence of inflammation in the initial period of implantation, and often cause peri-implant mucositis by dental caries and periodontitis because the surgical site easily contacts various bacteria in the oral environment [9]. In this aspect, it is expected that coating the implant surface with ginger will have an antimicrobial effect in specific oral bacteria [10] and will, thereby, improve the functionality of the implant material, as well as lower the percentage of failure after surgery. Extracting higher concentrations (in percentage) of the main compounds of natural product plays a very important role in promoting their pharmacological activities. In general, it was reported that the extraction efficiency of compounds can be improved through the application of various solvents in extraction and fractionation steps [11]. Consequently, in this study, the processes of citric acid extraction and n-hexane fractionation were applied in order to enhance extraction efficiency of the main compounds of ginger. Then, we aimed to maximize loading efficiency of the ginger_(fraction)_ on the surface of dental implants and to activate its biofunctionalities.

The ideal antibacterial agent in the oral cavity should have excellent selective antibacterial effects, only against the bacteria related to dental caries and periodontal disease, and low toxicity to the human body. In this respect, gelatin-methacrylic anhydride (GelMA) hydrogel has been actively used as a drug carrier in recent studies. The loading of the ginger_(fraction)_ on a GelMA hydrogel will be effective in maintaining the efficacy and safety of the hydrogel by inducing stable loading and sustained release of the ginger_(fraction)_. Photo-crosslinkable GelMA hydrogels are polymerized at a specific wavelength of light, so that the physical and chemical properties of the hydrogel can be controlled according to the amount of initiator, curing time, and light intensity [12,13]. It was judged that the characteristics (drug-loading concentration, thickness, gel density, etc.) of the coating layer of a ginger_(fraction)_-loaded hydrogel could be controlled by these factors [14]. In addition, due to the slow biodegradation of the GelMA hydrogel in the body, it is considered to be suitable for the sustained release of natural products. However, nonadhesive GelMA hydrogels form a local coating layer on the surface of titanium implants. Therefore, it was attempted to increase the adhesion of the ginger_(fraction)_-loaded GelMA hydrogel to the surface by improving the wettability of the titanium surface [15,16,17] and to induce the formation of a uniform coating layer.

The aim of this study was to enhance the biofunctionality and biocompatibility of a Ti dental miniscrew through the application of ginger. In order to increase the amount of ginger on the surface of the Ti screw and control its release, the loading of ginger in a GelMA hydrogel was considered. To improve the coating stability of the ginger hydrogel, the wettability of the Ti surface was modified through pre-calcification, and then the surface was coated with GelMA hydrogel containing different concentrations of the ginger_(fraction)_. Finally, through this coating technique, we were able to ensure the coating stability of the ginger_(fraction)_-loaded hydrogel and to improve the antibacterial effect, cell activity, and bone regeneration ability of the implant.

## 2. Materials and Methods

### 2.1. Materials

#### 2.1.1. Surface Modification (Pre-Calcification of TiO_2_ Nanotubes (TNC))

The specimens (10 × 10 mm) were prepared from pure titanium foil (0.1 mm thick, Kobe Steel Ltd., Kobe, Japan). After etching with 7 wt.% of HF and 12 wt.% of HNO_3_, the surface of pure Ti was premodified by sequential surface treatments (anodization, pre-calcification, and heat treatment). The surface was anodized in an electrolyte containing 1 wt.% NH_4_F and 79 wt.% glycerol (at 20 V for 1 h). The titanium specimen and platinum plate were connected to the anode and cathode of a constant DC power supply (Daunanotek Co., Ltd., Seoul, Republic of Korea), respectively. Pre-calcification was thermally cycled in NH_4_H_2_PO_4_ (0.05 M, 90 °C) and a saturated Ca (OH)_2_ (0.01 M, 100 °C) solution. The cycle was repeated 20 times in 1 min intervals. The specimens were then heated at 5 °C/min to 500 °C and held for 2 h.

#### 2.1.2. Fractionation of Ginger-Extract

The ginger_(fraction)_ was obtained through an extraction and fractionation process from the National Institute of Agricultural Sciences in Rural Development Administration as follows. Hot-air-dried ginger powder was mixed with 20 *w*/*v*% of the concentration in 70% ethanol containing 10 *w*/*v*% citric acid. It was then extracted using a stirrer at 80 rpm for 4 h at 60 °C, followed by filtration and concentration. The extract was then dissolved in water, and 95% n-hexane (C_6_H_14_) was mixed with a ratio of 2:3. It was fractionated for 48 h at 25 °C. The obtained n-hexane fraction was freeze-dried at −70 °C for solvent removal.

The main compounds (6-, 8-, 10-gingerols and 6-, 8-, 10-shogaols) in the ginger_(extract)_ and ginger_(fraction)_ were analyzed using ultra-performance liquid chromatography (UPLC; Nexera X2, Shimadzu, Kyoto, Japan) as reported previously [18].

#### 2.1.3. Synthesis of GelMA

Gelatin (Type A from porcine skin, Sigma-Aldrich, St. Louis, MO, USA), Dulbecco’s phosphate-buffered saline (DPBS), and methacrylic anhydride (MA) were used to fabricate GelMA. Powdered gelatin (10 *w*/*v*%) was completely dissolved in DPBS for 1 h at 60 °C. Then, 0.8 mL of MA per the weight (g) of gelatin was gradually added to the mixture. After stirring at 50 °C for 3 h, the substitution of functional groups between gelatin and MA was stopped by dilution with a 5-fold volume of DPBS. GelMA_(sol)_ was dialyzed against deionized water at 40 °C for 1 week using high-retention seamLess cellulose dialysis tubing with a molecular weight cutoff (MWCO) of 12,400 Da (flat width 40 mm) (Merck, Darmstadt, Germany). It was then freeze-dried at −80 °C for 5 days using a lyophilizer (FD8508, IlShin Lab Co., Ltd., Yangju-si, Republic of Korea).

### 2.2. Coating Technique of Ginger_(fraction)_-Loaded GelMA Hydrogel (GH) on TNC Surface

**Figure 1:**
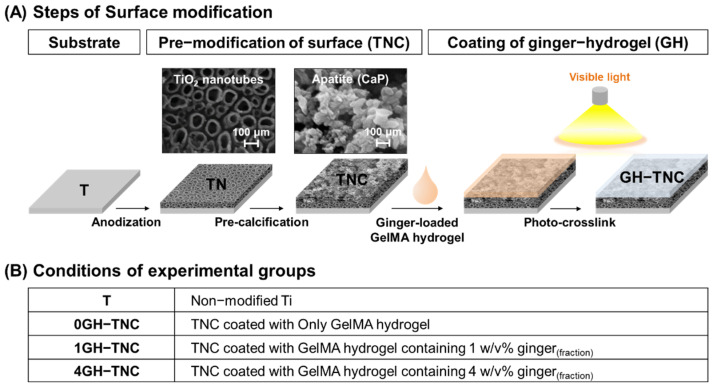
Description of the surface modification methods; (**A**) coating procedure and (**B**) group names.

The ginger_(fraction)_-loaded GelMA_(sol)_ was fabricated using freeze-dried GelMA, triethanolamine (TEA; Sigma-Aldrich, St. Louis, MO, USA), N-vinylcaprolactam (VC; Sigma-Aldrich, St. Louis, MO, USA), eosin Y disodium salt (Sigma-Aldrich, St. Louis, MO, USA), and the ginger_(fraction)_. It finally comprised 5 *w*/*v*% GelMA, 1.5 *w*/*v*% TEA, 1 *w*/*v*% VC, 0.5 mM eosin Y disodium salt, and different concentrations (0, 1, and 4 *w*/*v*%) of ginger_(fraction)_. Next, 25 µL of the ginger_(fraction)_-loaded GelMA mixture was coated on a 1 cm^2^ area of the TNC-modified surface. It was then photo-crosslinked by curing for 100 s using an LED light-curing unit (C02, Premium Plus, Bournemouth, UK) for polymerization into the hydrogel. The ginger_(fraction)_-loaded GelMA hydrogel (GH) on the TNC surface was freeze-dried at −80 °C for 3 days using a lyophilizer. The properties of GH−TNC surfaces were compared with that of the non-modified Ti surface. The groups were named according to the concentration (*w*/*v*%) of ginger_(fraction)_ in the hydrogel, as shown in Figure 1.

### 2.3. Surface Properties of Ginger_(fraction)_-Hydrogel Coated on TNC

The surface morphological changes of the groups were estimated by field-emission scanning electron microscopy (FE-SEM; SUPRA40VP, Carl Zeiss Co., Oberkochen, Germany) with energy-dispersive spectroscopy (EDS) capabilities. The crystalline structure of the surface was analyzed using a multi-purpose high-performance X-ray diffractometer (XRD; X’PERT-PRO Powder; PANalytical Co., Eindhoven, The Netherlands). XRD scanning was performed in the 2*θ* range from 10° to 90°. Fourier-transform infrared (FT−IR) spectroscopy (Spectrum GX, Perkin Elmer, Waltham, MA, USA) was performed at the Center for University-wide Research Facilities (CURF) of Jeonbuk National University to determine the chemical bonding properties of different ginger_(fraction)_ hydrogels on the surface at a resolution of 4 cm^−1^ in the range 500–4000 cm^−1^.

### 2.4. Release of Ginger_(fraction)_ from GelMA Hydrogels

Next, 25 µL of ginger hydrogels, the same as the volume coated on the surface, were fabricated and freeze-dried. Four samples were prepared per group for the release test. The sample was immersed in 500 µL of distilled water (DW) for 60 days. The amount of ginger_(fraction)_ was measured by absorption spectroscopy using a UV−vis Spectrophotometer (V-630; JASCO, Tokyo, Japan) after 15 days of immersion. The morphology of the ginger_(fraction)_ hydrogels with degradation was observed after 1, 5, 15, 30, and 60 days of immersion.

### 2.5. Antibacterial Test

#### 2.5.1. Suspension of Oral Strains

*Streptococcus mutans* (*S. mutans,* KCTC3065) and *Porphyromonas gingivalis* (*P. gingivalis*, KCTC5352) obtained from the Korean Collection for Type Cultures (KCTC) were used as representative oral strains for bacterial growth assays. The inocula pre-cultured on a sheep blood agar plate (BAP; ASAN Pharmaceutical, Seoul, Republic of Korea) were suspended at 1.5 × 10^7^ colony forming units (CFU)/mL in brain heart infusion (BHI; Difco, Becton Dickinson and Company, Sparks, MD, USA) broth.

Conditions for the incubation of bacteria are as follows. *S. mutans* was grown in a 5% CO_2_ incubator for 24 h at 37 °C. *P. gingivalis* was cultured for one week at 37 °C under anaerobic conditions using a GENbox jar (96127; BioMérieux SA, Marcy-l’Etoile, France) in the GENbox anaer system (96124; BioMérieux SA, Marcy-l’Etoile, France)

#### 2.5.2. Minimum Inhibitory Concentration (MIC) and Minimum Bactericidal Concentration (MBC) of Ginger_(fraction)_

The fractionated ginger solutions based on BHI were prepared according to the broth microdilution method from 10% to 0.01%. Diluted samples (100 μL) were mixed with a suspension of each strain (100 μL). Before and after incubation with bacteria under each incubation condition, absorbance was measured at a wavelength of 600 nm using an ELISA microplate reader (EMax® Precision Microplate Reader; Molecular Devices, Sunnyvale, CA, USA). The lowest concentration with an absorbance gap smaller than 0.01 was determined as the MIC. To determine the MBC, 100 μL aliquots extracted from samples higher than the MIC were spread in BAP and incubated as described in the MIC test. The MBC was determined by confirming the lowest concentration without visible bacterial growth on the BAP.

#### 2.5.3. Antibacterial Effect of the GH−TNC Coating Surface

The antibacterial effect of the ginger_(fraction)_-hydrogel coating was determined based on bacterial growth (%) on the surface. Five hundred microliters of each suspension was added to a 24-well plate with ginger_(fraction)_-hydrogel-coated surfaces. After incubating the bacteria at each condition, 100 μL aliquots were taken from each group and their absorbance was measured using an ELISA microplate reader at a wavelength of 600 nm. Bacterial growth was calculated as follows:

Viability (%) = Absorbance _(Experimental group)_ / Absorbance _(Control group)_ × 100 (1)

To observe the morphology of bacteria by scanning electron microscopy (SEM; JSM-5900, JEOL, Tokyo, Japan), the bacteria grown on the surface were fixed in 25% glutaraldehyde for 2 h. After washing with phosphate-buffered saline (PBS), staining was performed with a 1% osmium tetroxide solution for 2 h. The sample was then gradually dehydrated in 30%, 50%, 70%, 80%, 90%, 95%, and 100% of ethanol for 10 min.

### 2.6. Immersion Test in a Simulated Body Fluid (SBF)

Hanks’ balanced salt solution (HBSS; H2387, Sigma-Aldrich, St. Louis, MO, USA) adjusted to pH 7.4 was prepared for immersion tests in SBF. Specimens were placed at 37 °C in HBSS. The HBSS was refreshed every 2 days. After 10 days of immersion, the surface properties were characterized using FE-SEM with EDS and XRD.

### 2.7. Cytotoxicity Test

To examine the cytotoxicity of the GH−TNC coating on osteoblasts, mouse osteoblast cells (MC3T3-E1) obtained from American Type Culture Collection (Manassas, VA, USA) was used for the test. The medium for culture was prepared by adding 10% fetal bovine serum (FBS; Gibco Co., Waltham, MA, USA), 500 U/mL penicillin (Gibco Co., Waltham, MA, USA), and 500 U/mL streptomycin (Gibco Co., Waltham, MA, USA) to α-Minimum Essential Medium (α-MEM; Gibco Co., Waltham, MA, USA). After pre-culturing, a cell suspension was prepared at a density of 5 × 10^4^ cell/mL in the media. The non-coated and GH−TNC-coated samples (1 cm^2^) were immersed in culture media and incubated for 72 h at 37 °C and 5% CO_2_ to extract their main compounds. The extraction volume and conditions were in accordance with the EN ISO 10993−12:2004 requirements [19]. The media extracted from the groups were replaced after the cells from the suspension were attached in normal media. After 2 and 4 days of culture, the medium was replaced with fresh medium containing water-soluble tetrazolium salt (WST)-8 reagent from Cell Counting Kit-8 (Enzo Life Sciences Inc., Farmingdale, NY, USA). After incubation for 1.5 h, media absorbance was measured at 450 nm using an ELISA microplate reader. At the incubation time points, the cells were fixed in a mixture of 0.2% glutaraldehyde and 3% formaldehyde in PBS. The cells were then stained in PBS containing 0.3% crystal violet. An optical microscope (DM2500M; Leica, Wetzla, Germany) was used to observe the cell morphology.

### 2.8. In Vivo Test

#### 2.8.1. Implantation of Screw in Tibial Defect of a Rat

**Figure 2:**
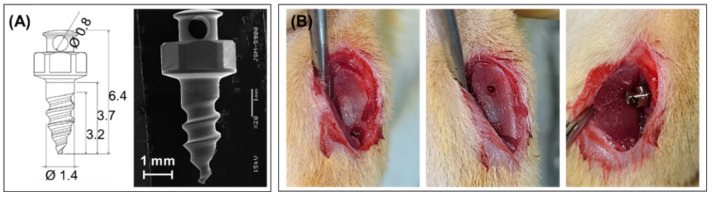
Implantation of a surface-modified Ti screw in the tibial defect; (**A**) design of the Ti screw, (**B**) procedure of the surgery.

This study was conducted in compliance with the Declaration of Helsinki and was approved by the Institutional Animal Care and Use Committee of the Jeonbuk National University Laboratory Animal Center (JBNU 2022-080). The screw for the in vivo test was designed, as shown in Figure 2A. Screws were implanted on both sides of the tibia of the rats. Next, 24 male Sprague Dawley rats (8 weeks old, 270–280 g weight) were used for implantation in the GH−TNC groups (*n* = 6). Rats were anesthetized by intraperitoneal injection of 0.6 mL/kg of tiletamine plus zolazepam (Zoletil 50, Virbac Laboratories, Carros, France) and 0.4 mL/kg of xylazine hydrochloride (Rompun, Bayer Korea, Seoul, Republic of Korea). After making a 1 cm incision over the tibia, a hole was drilled in the cortical bone using a contra-angle hand-piece (X-smart Endodontic Motor, Dentsply Maillefer, Switzerland) equipped with a 1.0-mm pilot round-headed burr (H1.316-018, Komet, Germany). The screw was then tightened using a self-tapping process (Figure 2B). Antibiotics (0.6 mL/kg) were administered subcutaneously (Amikacin, Samu Media Co., Ltd., Yesan-Gun, Republic of Korea) after surgery. Twelve rats were euthanized at each time point (2 and 6 weeks) and their tibial blocks were harvested.

#### 2.8.2. Histological Observations

The bone blocks harvested after two and six weeks of implantation were fixed in a 10% formalin solution. After 3 days of immersion in Villanueva Osteochrome Bone Staining solution (Polysciences, Inc., Warrington, PA, USA), the samples were dehydrated with increasing concentrations of ethanol (from 80% to 100%). The hard tissues were embedded in methylmethacrylate (Yakuri Pure Chemical Co., Kyoto, Japan), which was polymerized by adding benzoyl peroxide (Sigma-Aldrich, St. Louis, MO, USA). For histological observation using an optical microscope, the samples were sectioned and mounted on slides.

### 2.9. Statistical Analysis

One-way analysis of variance with Tukey’s test was used for statistical analysis of the results. Statistical significance was set at *p* < 0.05.

## 3. Results

### 3.1. Chemical Properties and Antibacterial Efficiency of Main Compounds in Ginger_(fraction)_

**Figure 3:**
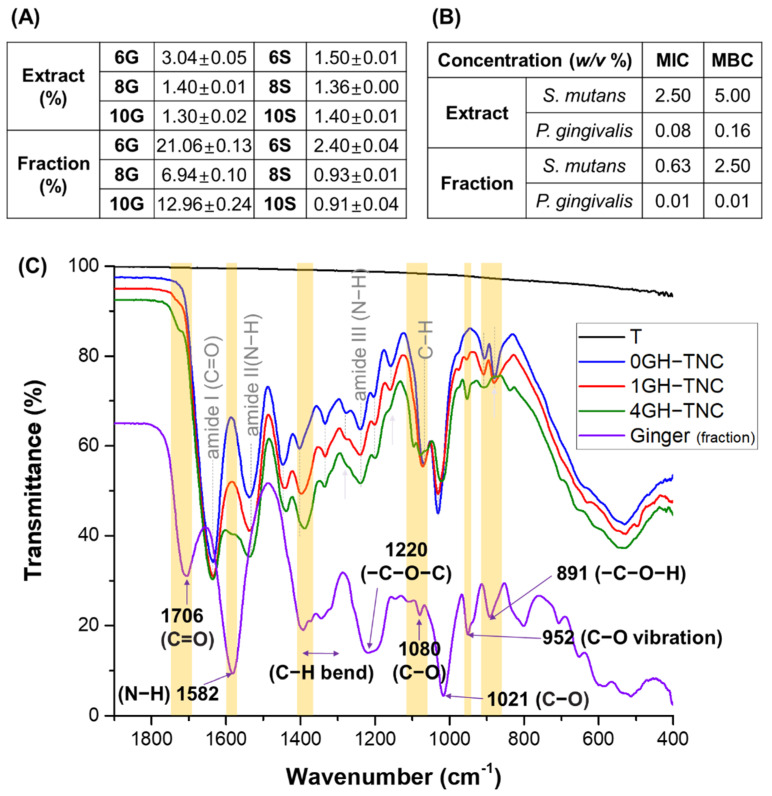
Characterization of ginger_(sol)_ [extract vs fraction]; (**A**) content of main compounds (%), (**B**) MIC and MBC against *Streptococcus mutans* and *Porphyromonas gingivalis*], and (**C**) chemical structure with the concentration of ginger_(fraction)_ in the GelMA hydrogel coating on the TNC surface.

The main compounds (6-, 8-, and 10-gingerols, and shogaols) in ginger were detected after extraction and fractionation. However, the content (%) of 6- and 10-gingerols in the total amount of solution increased upon fractionation with n-hexane after extraction compared to that upon extraction with acid-mixed ethanol alone (Figure 3A). Both the extract and the fraction showed excellent antibacterial effects against the representative oral strains (*S. mutans* and *P. gingivalis*) as indicated by comparing the antibacterial activity (MIC and MBC) (Figure 3B). The fraction showed superior growth inhibition and antimicrobial effects as indicated by lower MIC and MBC values.

In the FT-IR spectra (Figure 3C), the 0GH−TNC group showed clear peaks related to the GelMA hydrogel at 3079 cm^−1^ (tensile vibration of the C−H bond in amide B), 2940 cm^−1^ (C−H stretching vibrations), 1635 cm^−1^ (strong vibration of the C=O bond (amide I)), 1537 cm^−1^ (C−N and N−H bending (amide II)), and 1239 cm^−1^ (N−H bending (amide III)). In the 1GH−TNC and 4GH−TNC groups, the intensity of peaks related to the ginger_(fraction)_ became strong depending on the increase in concentration. The position of the peaks in the graph is marked in yellow.

### 3.2. Release Kinetics of Ginger_(fraction)_ from the GelMA Hydrogel

**Figure 4:**
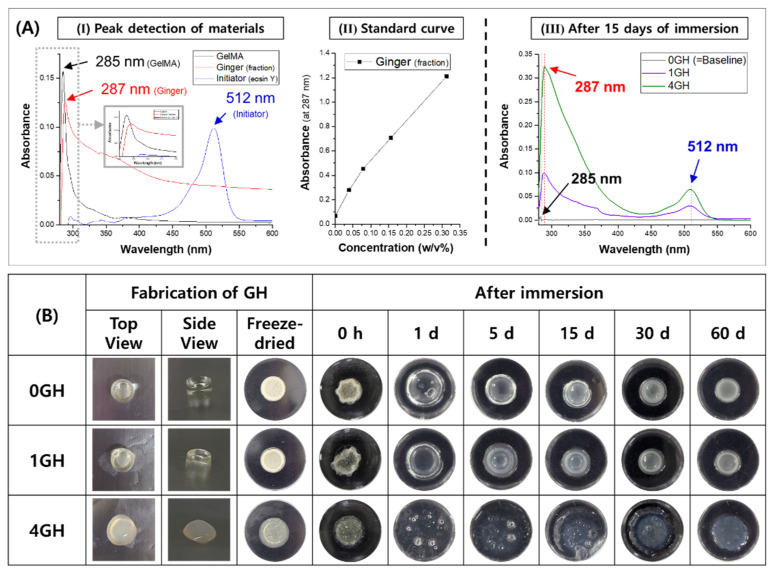
(**A**) Analysis of the ginger_(fraction)_-loaded hydrogel by UV−vis [(**I**) peak-detection of materials, (**II**) standard curve of absorbance with the concentration of ginger at 287 nm, (**III**) release of ginger from the hydrogel at 15 days of immersion in DW], and (**B**) degradation of ginger_(fraction)_-loaded hydrogels depending on time.

UV−vis measurements of the main materials used in the hydrogel manufacture confirmed that the maximum absorbance values for the GelMA, ginger_(fraction)_, and initiator (eosin Y) were detected at wavelengths of 285, 287, and 512 nm, respectively. The standard curve for ginger_(fraction)_ was obtained as shown in Figure 4A after measuring the absorbance with the standard concentrations of ginger_(fraction)_ at 287 nm. The release amount (mg) of ginger_(fraction)_ from the hydrogel was calculated after 15 days of immersion because the initial 2 weeks are reported to be critical for the success of implantation. The total amounts of the ginger fraction loaded in 25 μL of 1GH and 4GH hydrogels were 1 and 2 mg, respectively. During approximately 2 weeks, the 1GH and 4GH groups released 27.2% and 43.5% of ginger_(fraction)_ vs. the total amount, respectively. As shown in Figure 4B, a harder hydrogel was fabricated after polymerization at the lower concentration of ginger_(fraction)_ loading.

After immersion of the freeze-dried GH groups for 60 days, all groups rapidly absorbed the aqueous solution within 1 day, and the swelling of the hydrogel increased with an increase in the concentration of ginger_(fraction)_ loading. Degradation during the immersion period in the groups slowly progressed in the strongly polymerized hydrogel.

### 3.3. Characterization of GH−TNC Coating with the Concentration of Ginger_(fraction)_

**Figure 5:**
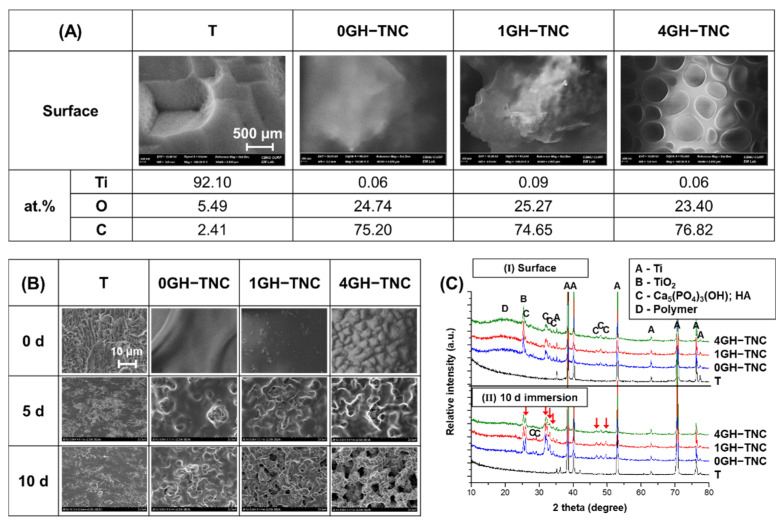
Characterization of the GH−TNC surfaces (**A**,**C-I**) before and (**B**,**C-II**) after 10 days of immersion in HBSS; the morphologies and composition were obtained by FE-SEM and the crystal structure by XRD.

After GH−TNC coating, the final surface only showed an increase in the concentration of C and O ions owing to the thick polymer layer. However, the XRD results in Figure 5C-I confirmed that crystalized TiO_2_ (anatase) and Ca_5_(PO_4_)_3_(OH) (hydroxyapatite; HA) were present on the surface after pre-modification. There was no phase transformation of the crystal structures in any of the GH−TNC groups. Although the coverage of TNC surface by the hydrogel was observed in all groups, the morphology of the surface mainly depended on the characteristics of the ginger hydrogel (Figure 5A). In the 0GH−TNC group, the top layer was coated with a dense polymer without any pores. However, as the concentration of ginger_(fraction)_ increased, a uniform and porous coating layer was observed.

Upon immersion of the GH−TNC surfaces in SBF, the GH layer slowly degraded as the immersion period progressed (Figure 5B). As the coating layer became thinner, the morphology of the pre-modified TNC surface was revealed. The degradation rate of the hydrogel rapidly increased depending on the concentration of the ginger_(fraction)_. Although the T group did not show crystallization of HA after 10 days of immersion in SBF, an increase in precipitation for the HA phase was observed on the surface in all GH−TNC groups, regardless of ginger_(fraction)_ addition (indicated by the red arrows in Figure 5C-II). After GH−TNC coating, the final surface only showed an increase in the concentration of C and O ions owing to the thick polymer layer. However, the XRD results in Figure 5C-I confirm that crystalized TiO_2_ (anatase) and Ca_5_(PO_4_)_3_(OH) (hydroxyapatite; HA) were present on the surface after pre-modification. There was no phase transformation of the crystal structures in any of the GH−TNC groups. Although coverage of the TNC surface by the hydrogel was observed in all groups, the morphology of the surface mainly depended on the characteristics of the ginger hydrogel (Figure 5A). In the 0GH−TNC group, the top layer was coated with a dense polymer without any pores. However, as the concentration of ginger_(fraction)_ increased, a uniform and porous coating layer was observed.

Upon immersion of the GH−TNC surfaces in SBF, the GH layer slowly degraded as the immersion period progressed (Figure 5B). As the coating layer became thinner, the morphology of the pre-modified TNC surface was revealed. The degradation rate of the hydrogel rapidly increased depending on the concentration of the ginger_(fraction)_. Although the T group did not show crystallization of HA after 10 days of immersion in SBF, an increase in precipitation for the HA phase was observed on the surface in all GH−TNC groups, regardless of ginger_(fraction)_ addition (indicated by the red arrows in Figure 5C-II).

### 3.4. Antibacterial Effect on GH−TNC Coating

The GH−TNC groups with the ginger_(fraction)_ (0, 1, and 4%) showed a decrease in bacterial viability by approximately 90%, 60%, and 5% or less for all bacteria, respectively. The 0GH−TNC showed a lower bacterial viability (%) than that of the non-coated group, but its effect was much weaker than that of the ginger-loaded groups. All GH−TNC groups treated with the ginger_(fraction)_ showed a significant growth inhibitory effect on both *S. mutans* and *P. gingivalis*. Further, antibacterial activity increased depending on the concentration of the ginger_(fraction)_.

**Figure 6:**
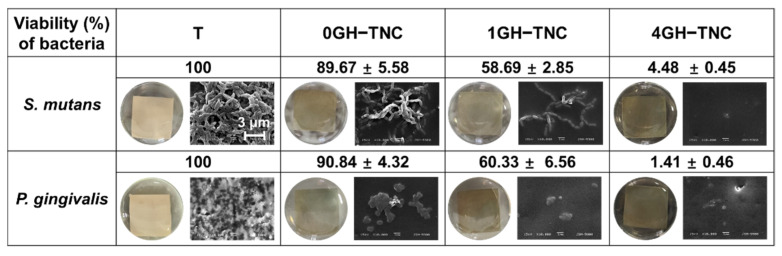
Bacterial viability (%) and morphology on the GH−TNC surfaces for *Streptococcus mutans* and *Porphyromonas gingivalis*.

Morphological observations of bacterial growth on the surface (Figure 6) confirmed that biofilms were formed on the surface in the T and 0GH−TNC groups. In the other GH−TNC groups, the number of attached *S. mutans* chains decreased as the concentration of ginger-loaded hydrogel on the surface increased. In the culture of *P. gingivalis*, the population of bacterial colonies rapidly decreased at higher concentrations of the ginger_(fraction)_.

### 3.5. Cytotoxicity of the GH−TNC Coating

**Figure 7:**
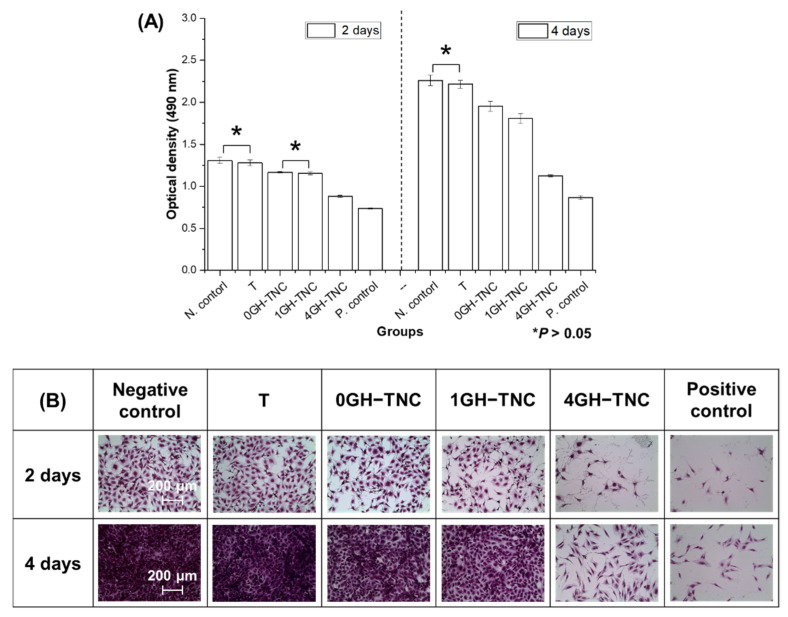
(**A**) Proliferation of MC3T3-E1 cells in the extract media from the modified surfaces and (**B**) their morphologies. * It means that there is not significant difference (* *p* > 0.05).

After 2 and 4 days of osteoblast culture, only the non-coated group showed no significant difference from the negative control (*p* > 0.05) (Figure 7). Higher concentrations of ginger_(fraction)_ in the GH−TNC groups significantly decreased cell proliferation. On the fourth day of culture, the 4GH−TNC group showed a low cell viability of approximately 50% compared with that of the negative control for all cells, and the possibility of cytotoxicity was confirmed. However, except for the 4GH−TNC group, all GH−TNC groups were compatible with osteoblasts as they showed more than 70% cell viability.

### 3.6. Bone Regeneration by GH−TNC Coating for an In Vivo Model

**Figure 8:**
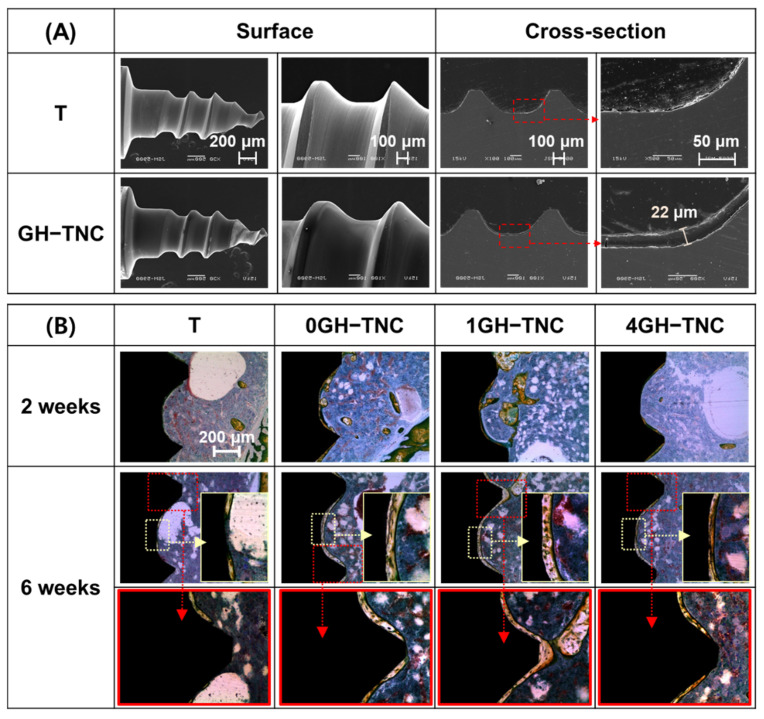
(**A**) SEM images on the surface and cross-section after GH−TNC coating on the Ti screw; (**B**) histological images after implantation of the surface-modified screw on the tibial defect of a rat for 2 and 6 weeks.

With the GH−TNC coating on the surface of the mini-screw, a uniform and smooth hydrogel coating layer with a thickness of approximately 22 μm was formed on the surface, as shown in Figure 8A. During the initial 2 weeks of implantation, there was no significant difference in bone formation around the surface between the experimental groups and the control group. However, 6 weeks after implantation, osteocytes were concentrated around the surface of all GH−TNC groups, and remarkable bone growth was observed. The thickness of the new bone showed a tendency to decrease according to the concentration of the ginger_(fraction)_, but all GH−TNC groups induced superior bone growth compared to that in the control group. In all groups, new bone grew more rapidly in the groove of the screw thread than in the ridge (Figure 8B). The control group showed non-uniform bone regeneration in parts of both the ridge and groove of the screw thread, whereas the 1GH−TNC group showed thick and uniform bone formation in all parts.

## 4. Discussion

Recently, various studies have been conducted on the medical application of natural products [20,21,22,23]. In this study, to improve the functionality of dental implants using ginger_(fraction)_, the ginger was combined with pre-modification using TNC and the photo-crosslinkable GelMA hydrogel. Stable loading and release of the ginger_(fraction)_ on the surface were expected.

In general, it is known that the extraction of natural products in acid-based solvents can increase extraction efficiency [24] and is human-friendly [25]. Additional fractionation of extracts by organic solvents, such as hexane, chloroform, and ethyl acetate, etc., is effective for improving the yield of natural products [26,27]. As shown in Figure 3A,C, ginger fractionated using n-hexane showed higher contents of polyphenolic and flavonoid compounds than those in unfractionated ginger [28,29,30], contributing to the relatively high content of 6- and 10-gingerols. It was reported that those phenolic and flavonoid compounds in ginger inhibit bacterial growth by inhibiting a function of cytoplasmic membrane and by interrupting an interaction between cell membrane and mitochondria in bacteria [31,32,33]. The concentration of gingerol (6-gingerol), a phenolic phytochemical compound, is related to the antibacterial activity of plant extracts [34]. The n-hexane fraction containing a higher content of phenolic compounds (6-gingerol) showed lower MIC and MBC values against two types of oral bacteria than those of the extracts, indicating excellent antibacterial activity [35]. However, some researchers [36,37] have suggested that it may have negative toxic effects at high doses, as they observed dose-dependent toxicity of 6-gingerol, along with its strong antibacterial effect. Therefore, for applying the ginger_(fraction)_ in the body, controlling the released dose is a very important factor that needs to be considered.

To address this concern, we attempted to reduce the toxicity caused by the rapid release of the ginger_(fraction)_ by controlling the release concentration of the ginger_(fraction)_ by loading it inside a hydrogel. The biodegradability of the hydrophilic GelMA hydrogel can be controlled using various manufacturing factors. Further, it has excellent biocompatibility and is often used as a carrier for drug delivery [38]. It is mainly effective in inducing the sustained release of drugs [39], and has proven biosafety and an excellent ability for bone regeneration [40,41]. To improve the adhesive property in coating the ginger-loaded GelMA hydrogel on the surface, pre-modification (TNC: anodization and pre-calcification) was performed on a pure Ti surface. As a result, a flower-like surface with crystal structures of TiO_2_ and Ca_5_(PO_4_)_3_(OH) was formed on the Ti surface. The formation of these crystalline phases was effective in modifying the surface to be super-hydrophilic as it activated the -OH functional groups on the surface compared to those with non-modified Ti [42,43]. As the surface wettability increased, the modified TNC surface rapidly absorbed the hydrophilic hydrogel, thereby increasing the surface adhesion of the coating layer. Further, it did not transform the crystal structures formed by surface modification and helped to form a uniform hydrogel film without local concentration of hydrogel at the center after photo-crosslinking.

Because the ginger _(sol)_ obtained by acid extraction and n-hexane-fractionation was lipid-soluble [44], it was expected to show low solubility when mixed with a water-soluble GelMA hydrogel. In fact, addition of the ginger_(fraction)_ disturbed photo-crosslinking between GelMA and the initiator (eosin Y) for polymerization. Based on Figure 4B and Figure 5A, the density of polymerization and the stability of the coating decreased with the added concentration of the ginger_(fraction)_. A pore-like boundary was observed in the freeze-dried hydrogel layer on the surface. In the FT-IR results of the ginger_(fraction)_ hydrogel coated on the TNC surface, the peak intensity related to phenolic compounds of gingerol increased depending on the concentration of the fraction loaded in the hydrogel, but the peak intensity related to the crosslinking of the GelMA hydrogel decreased.

The amount of ginger_(fraction)_ released from the hydrogel coating was estimated after 15 days of immersion in DW. The ginger_(fraction)_-loaded hydrogels showed a release of approximately 27% and 43% with increasing the loading concentration, and the release rate of ginger_(fraction)_ was rapid as the concentration was high. The low density of polymerization by the addition of ginger accelerated the degradation of the hydrogel upon immersion in the aqueous solution, which hastened the release of ginger_(fraction)_. Despite adding a high concentration of ginger, none of the hydrogel groups released the total amount of ginger from the hydrogel until 15 days. Moreover, degradation of the hydrogel was sustained for approximately 60 days or more. Based on this, it was predicted that when the ginger_(fraction)_ hydrogel was coated on the surface of the TNC-modified Ti implant, the ginger fraction would be released continuously for a period of approximately 2 months with the slow degradation of the GelMA hydrogel.

To verify the antibacterial effectiveness of the final GH−TNC coating layer, two representative oral bacteria were grown, and their activity was observed. As a result, the hydrogel-coated TNC surface showed a very low growth inhibitory effect compared to that of other GH−TNC groups and led to the formation of a wide biofilm. All the GH−TNC surfaces with the ginger_(fraction)_ effectively inhibited the growth of both bacteria. However, the GH−TNC group with the highest concentration of the ginger_(fraction)_ showed maximum 95% and 98% antibacterial effects against *S. mutans* and *P. gingivalis* with the loading concentration of the ginger_(fraction)_. This meant that the ginger_(fraction)_ released from the GH−TNC surface effectively inhibited the adhesion of bacteria. The main phenolic compounds in ginger_(fraction)_, such as gingerols and shogaols, interfered with the bacterial physiology [45,46].

To confirm biocompatibility according to the concentration of the ginger_(fraction)_ in the hydrogel, the bioactivity, cytotoxicity, and biosafety of the hydrogels were evaluated. In general, the anatase phase of TiO_2_ obtained by anodization induces the formation of OH- groups on the surface and creates sites for the nucleation of hydroxyapatite in the interaction with SBF. Further, TNC surfaces prepared by pre-calcification are known to exhibit excellent bioactivity in SBF [47]. The HA crystal structure formed on the surface continued to absorb and grow both calcium and phosphate in SBF [48]: 10Ca^2+^_(aq)_ + 6PO_4_^3−^_(aq)_ + 2OH^−^_(aq)_ ↔ Ca_10_(PO_4_)_6_(OH)_2(s)_. Through these reactions, it was confirmed in Figure 5C that the TiO_2_ peak was reduced in all the GH−TNC groups, and that the crystallization of HA was accelerated. Loading a high concentration of ginger_(fraction)_ induced a relatively low intensity of HA precipitation. However, it still showed a better effect on bioactivity in SBF than in the control group. Therefore, the existence of a TNC layer in the GH−TNC groups was found to be more effective at supplementing the decrease in bioactivity by the addition of ginger_(fraction)_.

For application in dental implants, the prevention of inflammation, as well as the maintenance of osteointegration at the surgical site are critical during the initial healing period. Osteoinductivity of the implant surface is one of the most important factors for the success of surgery. It has been reported that some compounds obtained during the extraction and fractionation of natural products may reduce osteoblast proliferation depending on their concentration. Many researchers have mentioned the necessity of considering potential toxicity to osteocytes. In a previous study [49], cytotoxicity was still observed at very low concentrations as the 50% inhibitory concentration of the ginger extract and fraction on L929 cells after 2 days of culture was 101.0 µg/mL and 87.28 µg/mL, respectively. However, in Figure 7, 50% or higher cell viability was found to be maintained in all GH−TNC groups, including the 4GH−TNC group with the highest concentration of the ginger_(fraction)_ (the total amount of ginger_(fraction)_ present on the 4GH−TNC surface: 1 mg/cm^2^). In addition, during the 4 days of culture, the groups other than the 4GH−TNC group showed cell viability higher than 70%, proving its safety for osteoblasts. The phenolic compounds, gingerol and shogaol, are among the main compounds in ginger that can limit osteoblast proliferation [50]. For this reason, the group treated with a high concentration of the ginger_(fraction)_ showed a decrease in cell growth. Although loading the ginger_(fraction)_ in the hydrogel proved the possibility of using a higher concentration by inducing slow release compared to the direct use of ginger_(fraction)_, loading ginger_(fraction)_ at less than 4 *w*/*v*% in the GH−TNC coating is safer according to ISO 10993-5:2009 [51].

The bone-regenerative effect of the implant screw with the GH−TNC-coating was compared in vivo. A delay was still observed in bone regeneration depending on the concentration of the ginger_(fraction)_, such as in the results of the in vitro cell test. The hydroxyapatite accelerates bone mineralization by increasing the proliferation and differentiation of osteoblasts. GelMA hydrogels are effective for drug delivery, as well as new bone formation, by promoting the proliferation of osteoblasts through the geometrical structure of the hydrogel [40]. For these reasons, the GelMA hydrogel-coated TNC group without the ginger_(fraction)_ showed the fastest and most effective osteosis formation. Moreover, the GH−TNC-coated implant surfaces also induced excellent bone regeneration by forming a thick and uniform osteosis layer compared with that of the untreated implant surface. The ability for osteosis formation was highly supplemented by the combination of both carrier (GelMA hydrogel) and TNC coating compared to non-treated group, although it was still reduced with the concentration. Some researchers reported that the modified growth of the cells was led as the functional polymer was combined for use of specific nanoparticles in the biomedical application, suggesting a new platform for tissue engineering [52,53]. This means that the promotion of bone formation by a combination of both GelMA hydrogel and TNC coating can be preferentially contributed to by improvement in the bone regeneration. Therefore, this coating layer was sufficient to overcome the disadvantages of dose-dependent toxicity of the ginger_(fraction)_ and to improve the biofunctionalities of Ti implants.

## 5. Conclusions

Various compounds derived from ginger have been used in medical applications owing to their excellent antibacterial properties, low toxicity in the body, and pharmacological activity. However, some researchers have reported these advantages as well as the potential toxicity of risk factors that may be generated in the process of extracting and fractionating these natural products. In this study, fractionation of ginger extract using n-hexane was very useful to obtain a higher amount of 6- and 10-gingerols from the extract. Gingerol, an aromatic compound, has excellent antibacterial activity against specific bacteria, such as *Streptococcus mutans* and *Porphyromonas gingivalis*, which are associated with dental caries and periodontitis. However, excessive concentrations of ginger induce a dose-dependent decrease in the proliferation of osteoblastic cells.

In this aspect, the implant coating method of loading the ginger_(fraction)_ in the GelMA hydrogel can successfully sustain the release of ginger rather than directly applying the ginger extract. Before coating the ginger-loaded hydrogel, the surface was modified by anodization and pre-calcification to obtain a super-hydrophilic surface, with TiO_2_ and hydroxyapatite formed on the surface. The modified surface improved the coating capabilities (adhesion and uniformity) of the functional ginger hydrogel and enhanced the bioactivity of the Ti surface in SBF. As the ginger hydrogel increased the efficiency of ginger-loading and continually released the main compounds in ginger, it induced a synergistic effect on the inhibition of bacterial adhesion and an increase of osteogenesis around the Ti surface. This means that it can show excellent effects in preventing oral diseases and promoting bone regeneration simultaneously on the bone defect upon Ti implantation.

Finally, it was concluded that the GH–TNC coating method must be the effective method to give superior functionalities in terms of the antibacterial activity, bioactivity, biocompatibility, and bone regeneration on the surface of Ti implant. However, it was confirmed that the bone was slowly regenerated by the decrease in the initial proliferation of osteoblast when the loading of an excessive concentration of ginger fraction even in hydrogel. Therefore, determining an appropriate concentration of ginger in clinical applications will be the most important factor to acquire the biosafety together with superior biofunctionalities on the surface of dental implant. Moreover, this study suggested that the dose-dependent toxicity of natural products can be controlled using the release capacity by the biodegradability of the carrier, being meaningful for expanding the application fields of functional natural products and various biodegradable materials.

## Data Availability

Data are available upon request from the authors.
